# Preclinical and Clinical Studies Demonstrate That the Proprietary Herbal Extract DA-5512 Effectively Stimulates Hair Growth and Promotes Hair Health

**DOI:** 10.1155/2017/4395638

**Published:** 2017-04-30

**Authors:** Jae Young Yu, Biki Gupta, Hyoung Geun Park, Miwon Son, Joon-Ho Jun, Chul Soon Yong, Jeong Ah Kim, Jong Oh Kim

**Affiliations:** ^1^Dong-A Pharm Research Institute, Pharmaceutical Product Research Laboratories, Yongin 449-905, Republic of Korea; ^2^College of Pharmacy, Yeungnam University, 214-1 Daedong, Gyeongsan 712-749, Republic of Korea; ^3^College of Pharmacy, Research Institute of Pharmaceutical Sciences, Kyungpook National University, Daegu 41566, Republic of Korea

## Abstract

The proprietary DA-5512 formulation comprises six herbal extracts from traditional oriental plants historically associated with therapeutic and other applications related to hair. Here, we investigated the effects of DA-5512 on the proliferation of human dermal papilla cells (hDPCs) in vitro and on hair growth in C57BL/6 mice and conducted a clinical study to evaluate the efficacy and safety of DA-5512. DA-5512 significantly enhanced the viability of hDPCs in a dose-dependent manner (*p* < 0.05), and 100 ppm of DA-5512 and 1 *μ*M minoxidil (MXD) significantly increased the number of Ki-67-positive cells, compared with the control group (*p* < 0.05). MXD (3%) and DA-5512 (1%, 5%) significantly stimulated hair growth and increased the number and length of hair follicles (HFs) versus the controls (each *p* < 0.05). The groups treated with DA-5512 exhibited hair growth comparable to that induced by MXD. In clinical study, we detected a statistically significant increase in the efficacy of DA-5512 after 16 weeks compared with the groups treated with placebo or 3% MXD (*p* < 0.05). In conclusion, DA-5512 might promote hair growth and enhance hair health and can therefore be considered an effective option for treating hair loss.

## 1. Introduction

Alopecia or hair loss is a dermatologic disease, which affects men and women, and often affects self-esteem and personal attractiveness, potentially leading to depression and other negative effects [1]. Alopecia is caused by nutritional, autoimmune, or environmental factors [2–4]. A striking observation of people with alopecia is that, despite their poor quality of life, most patients do not pursue therapy, mainly because of the poor efficacy of available treatment approaches, the associated adverse effects, tolerance to treatment, and lack of proper information.

Minoxidil (MXD) is a potassium channel opener and vasodilator that is widely recommended to treat androgenetic alopecia, which increases the duration of “anagen” and enlarges miniaturized and suboptimal follicles. However, the mechanism of action of MXD, which stimulates hair growth, is not well understood [5–8]. Despite the perceived advantages of MXD for treating alopecia, it causes numerous dermatologic adverse effects such as scalp irritation, dryness, sealing, itching, erythema, and contact dermatitis [9–11].

Finasteride is a type-2 inhibitor of 3-oxo-5*β*-steroid 4-dehydrogenase (5*α*-reductase), which was studied as a treatment for male or female pattern baldness. Although there is limited evidence of the efficacy of finasteride, it may be considered a treatment option for patients who fail to respond to topical MXD [12]. Finasteride is well tolerated; and 2.5 mg daily seems to achieve better results compared with 1.0 mg daily. The adverse effects of finasteride include decreased libido and erectile dysfunction in men, and finasteride is contraindicated for treating pregnant women because of its teratogenicity [13]. Despite the efficacy of these drugs under certain circumstances, their long-term effects and side effects remain problematic. Therefore, researchers have intensified their interest in evaluating alternative remedies such as herbal medicines and products. Several herbal formulations are commercially available to treat alopecia, such as hair tonics, hair-growth stimulants, hair conditioners, hair-cleansing agents, and antidandruff agents [14].

The proprietary DA-5512 formulation includes herbal extracts from* Thea sinensis* L.,* Emblica officinalis*,* Pinus densiflora*,* Pueraria thunbergiana*,* Tribulus terrestris*, and* Zingiber officinale*. All sources are traditional oriental plants that have long been used for the therapy of ailments related to hair.* Thea sinensis* L. (green tea) has potential beneficial effects because of the anticancer and antioxidant properties of its component epigallocatechin-3-gallate (EGCG), a major constituent of polyphenols [15]. Moreover, EGCG promotes hair growth by stimulating proliferation and inhibiting apoptosis of hair dermal papilla cells [16].

*Emblica officinalis* (Indian gooseberry or Amla), which is an important herbal component of Thai traditional recipes, is believed to slow aging. Moreover, Amla acts as a diuretic, laxative, liver tonic, refrigerant, stomachic, restorative, alterative, antipyretic, anti-inflammatory, and hair tonic as well as an antiulcer, antidyspeptic, and digestive agent [17]. A fixed oil is obtained from these gooseberries, which is used to strengthen and promote hair growth. The dried fruit, which improves hair hygiene, has long been utilized as an important ingredient of shampoo and hair oil [18]. Thus, the Indian gooseberry is used as a hair tonic in traditional recipes for enriching hair growth and pigmentation.

*Phyllanthus emblica* L. is a potent inhibitor of 5*α*-reductase, which promotes the growth of the hair of C57BL/6 mice [19]. The pine needle (*Pinus densiflora* Sieb & Zucc.) is commonly used as a herbal medicine in East Asian countries. Pine needle tea is high in vitamin A, beta-carotene, and vitamin C, which prevent oxidative stress in the skin and help to keep skin looking young by eliminating free radicals. Vitamins A and C help prevent hair loss and dandruff as well [20].* Pueraria thunbergiana* (kudzu) is a climbing plant of the Leguminosae family, which is native to China, Japan, and Korea.

The roots and flowers of* P. thunbergiana* are used in traditional medicine because of their medicinal properties [21, 22], and a study of a murine model of hair loss demonstrates their antiandrogenic activity and proliferative effects on hair growth [19]. Similarly, the rhizome of the widely used member of the ginger family* Zingiber officinale* has been used for thousands of years to enhance the flavor and aroma of food. Further,* Z. officinale* rhizomes are a source of antimicrobials as well as nonsteroidal anti-inflammatory drugs [23]. Ginger is a powerful antifungal agent that is used in shampoo to treat dandruff [24]. The antihypertensive effects of fruits of* T. terrestris* are similar to those of MXD, and its beneficial effects are attributed in part to its ability to release nitric oxide from the endothelium and nitrergic nerve endings [25]; however, there are no reports of its effects on human hair growth. The present study was undertaken to investigate the pharmacological effects of DA-5512 on the proliferation of human dermal papilla cells (hDPCs) in vitro and its effects on C57BL/6 mice and to evaluate its efficacy and safety when administered to human subjects.

## 2. Materials and Methods

### 2.1. Materials

The plants listed in Table 1, which were purchased locally or from farms located in India and China, were ultrasonically extracted in 100% ethanol at room temperature for 3 days. The extracts were concentrated using a rotary vacuum evaporator (Eyela N-N, Tokyo, Japan), and the mixed concentrates were collected and sterilized for 10 min. The sterilized concentrates were filtered twice and then analyzed using high-performance liquid chromatography (HPLC) to determine their phytochemical contents as described in the next section.

### 2.2. Phytochemical Analysis

The HPLC apparatus (Infinity 1290 System; Agilent, Mississauga, Ontario, Canada) was equipped with an Eclipse Plus C_18_ column (150 mm × 2.1 mm id, 1.8 *μ*m; Agilent, Mississauga, Ontario, Canada). The column was eluted with a gradient formed by water and formic acid (0.1%) (solvent A) and acetonitrile (solvent B). The initial mobile phase contained 98% solvent A and 2% solvent B and was delivered for 10.0 min, and then solvent B was increased to 50% and then to 90% after 30.0 min and 40.0 min, respectively, and was then equilibrated for 10 min using the initial conditions. The flow rate was 0.35 mL/min, the column temperature was 40°C, the detection wavelength was 254 nm, and the injection volume was 1 *μ*L.

### 2.3. Effect of DA-5512 on the Proliferation of hDPCs

#### 2.3.1. Isolation and Culture of hDPCs

Human occipital scalp skin specimens from hair transplantation surgery were obtained after receiving the patients' informed consent. The Institutional Ethics Committee of Yonsei University, Wonju College of Medicine, Wonju, Korea, approved this study, which was conducted according to guidelines of the Declaration of Helsinki and Tokyo for humans. Samples were dissected into single HFs, and human dermal papillae were obtained from individually isolated HFs as previously reported [26, 27]. Isolated hDPCs were transferred to a plastic dish and cultured in Dulbecco's modified Eagle's medium (DMEM; Gibco BRL, Gaithersburg, MD, USA) supplemented with penicillin (100 IU/mL), streptomycin (100 *μ*g/mL), and 10% fetal bovine serum (FBS) (HyClone, Logan, UT, USA) in a humidified incubator set to 37°C that contained an atmosphere of 5% CO_2_. Second and third passage hDPCs were used.

#### 2.3.2. Viability Assay

Cell viabilities were determined using 3-[4,5-dimethylthiazol-2-yl]-2,5 diphenyl tetrazolium bromide (MTT) [28]. Briefly, 1 × 10^4^ cells in 100 *μ*L of growth medium were seeded into each well of a 96-well plate, allowed to adhere for 24 h, and then treated with serial doses of DA-5512 (0–10,000 ppm) or 1 *μ*M MXD (positive control) (Sigma-Aldrich, St. Louis, MO, USA) for 24 and 48 h, respectively [29]. After treatment, the medium in each well was removed and replaced with phosphate-buffered saline (PBS) containing 5 mg/mL MTT, and the plate was then incubated at 37°C for 4 h. The remaining supernatant was then removed, and 100 *μ*L of DMSO was added to each well and mixed thoroughly to dissolve the crystallized formazan. To ensure that all formazan crystals were completely dissolved after 10 min incubation, the optical density at 540 nm was determined using an ELISA reader. Cell viability (%) = (mean test absorbance)/(mean control absorbance) × 100.

#### 2.3.3. Proliferation Assay

Ki-67 expression was determined as a marker of cell proliferation. For this purpose, hDPCs were incubated on an 18 mm coverslip for 24 h in the presence of 100 ppm DA-5512 or 1 *μ*M MXD. The cells were then washed twice with PBS, fixed in 4% paraformaldehyde in PBS for 15 min at 37°C, permeabilized with 0.25% triton X-100 in PBS for 5 min, and blocked with 1% BSA in PBS for 30 min. The cells were incubated with an anti-Ki-67 antibody (1 : 100 dilution) (Abcam, Cambridge, MA, USA) overnight at 4°C, followed by incubation with Alexa Fluor 594-conjugated goat anti-rabbit IgG (1 : 200 dilution) for 1 h at room temperature. The hDPCs were then washed and counterstained with 1 *μ*g/mL DAPI for 5 min and mounted on a glass slide. Images of fluorescence were acquired using a confocal laser scanning microscope (TCS SPE; Leica Microsystems GmbH, Wetzlar, Germany).

### 2.4. Analysis of the Effect of DA-5512 on the Growth of Hair in C57BL/6 Mice

#### 2.4.1. Animals and Treatments

Seven-week-old male C57BL/6 mice were purchased from Daehan Biolink, Inc. (Eumseong, Korea). The animals were housed under conventional conditions that included a standard diet, ad libitum water, and a 12 h light cycle. Animals were first acclimatized for 1 week, and 20 mice were randomly separated into four groups of five mice each. Group 1, which served as the negative control (NC), was topically given 30% ethanol, and Group 2, which served as the positive control (PC), received 3% topical MXD (Minoxil®, Hyundai Pharm., Korea) hair tonic. Groups 3 and 4 received 1% and 5% topical DA-5512 hair tonic, respectively. The hair on the back of each mouse was shaved with an electric clipper, avoiding injury or stimulation of the skin, which was then followed by application of a depilatory cream to clean the remaining hair. The mice were then allowed to rest for 24 h, and 0.2 mL of the specified substance was then topically applied to the shaven areas daily for 14 days. Hair regrowth was examined and digital images were acquired using a Nikon Cool Pix P100 (Tokyo, Japan) on days 1, 7, 10, and 14. Changes in the area of hair regrowth (%) within the shaven area were evaluated using ZoomBrowser software (Canon, Japan). For determination of hair weight and length, 30 hairs were picked randomly from the shaven area of each mouse on day 14, and the weights and lengths of the hairs were recorded.

#### 2.4.2. Hematoxylin and Eosin (H&E) Staining

H&E staining was employed to determine the effects of DA-5512 on HFs. The mice were sacrificed upon completion of the study. Their dorsal skin was removed, fixed in 10% formaldehyde solution, and embedded in paraffin. Skin sections (2-3 *μ*m) were stained with H&E, and follicular lengths and numbers as well as follicular morphologies were observed using a light microscope (Olympus, Tokyo, Japan).

#### 2.4.3. Statistical Analyses of the Mouse Data

Statistical analyses were performed using one-way ANOVA followed by Dunnett's test. The data are reported as the mean ± SD.

### 2.5. Treatment of Hair Loss and Promotion of Scalp Health in Patients with Alopecia

#### 2.5.1. Study Subjects

The subjects included adults aged 20 to 60 years who were diagnosed with mild alopecia. Men and women were categorized according to the Norwood-Hamilton or Ludwig classifications, respectively. Participants showed no evidence of skin disorders, had no history of cardiovascular, renal, or hepatic disease, and did not admit to applying any topical treatment drug (steroids, cytotoxic agents, vasodilators, antihypertensives, anticonvulsants, *β*-blockers, and diuretics) or any of the following agents: spironolactone, cimetidine, diazoxide, cyclosporine, ketoconazole, or replacement hormones. Subjects were excluded if they underwent surgery for hair loss, such as a hair transplant. Pregnant and lactating women were excluded as well. The Institutional Review Board of Chung-Ang University Hospital approved this study (C2012223[918]), which was performed in the P&K Hair Clinic Center.

#### 2.5.2. Study Design

Changes in hair characteristics after 8 and 16 weeks of treatment were photographed and compared with baseline (Canon EOS 550D; Canon, Japan). An area of the scalp of each patient was defined before the first treatment and marked by a tattoo to locate the exact area after shaving. The investigator photographed the vertex and frontal area of the scalp. A randomized study design was utilized as follows: 5% topical DA-5512 (Dong-A Pharmaceutical Co. Ltd., Korea) (*n* = 8); 3% topical MXD (positive control; capillus, Dong-A Pharmaceutical, Korea) (*n* = 7); or placebo (vehicle, 30% ethanol) (*n* = 8). Patients were given 1 mL of solution on the central scalp region twice daily at 12-h intervals for 16 weeks. The study comprised a baseline clinical visit (week 0) and two follow-up visits (weeks 8 and 16).

#### 2.5.3. Study Endpoints

The primary efficacy endpoint included changes in hair density (*n*/cm^2^), hair-shaft diameter (mm), and hair-growth rate (mm/day) among the three groups. Hair density and hair-shaft diameter were assessed using phototrichogram software (Folliscope 4.0, Lead M, Seoul, Korea) at baseline and at 8 and 16 weeks of treatment. The investigator's assessment scores for hair density of each group were assigned, and an investigator uninformed of the nature of the study used a standardized 7-point rating scale for evaluating the changes in hair density acquired from photographs of the patients' scalps at these times. The scores (±*n*) of the standardized 7-point rating scale were as follows: greatly decreased (−3), moderately decreased (−2), slightly decreased (−1), unchanged (0), slightly increased (+1), moderately increased (+2), and greatly increased (+3). The secondary efficacy endpoint was the comparison of changes according to the investigator's assessment and the patients' self-assessments of the efficacy of the treatment according to hair growth, hair density, or satisfaction. For the latter, a 10-point scale was used as follows: 0 (lowest) to 10 (highest), and a patient's self-assessment of scalp condition used a standard 5-point rating scale as follows: 0 (greatly decreased) to 5 (greatly increased).

#### 2.5.4. Safety

Safety monitoring was conducted through clinical evaluations and observations for adverse reactions experienced by any subjects.

#### 2.5.5. Statistical Analysis for Clinical Study

Changes in parametric data from the study were analyzed using analysis of variance (ANOVA), and *p* < 0.05 indicates statistical significance. The Wilcoxon signed rank test was used for nonparametric data obtained from patients' questionnaires after they used the products.

## 3. Results

### 3.1. Effects of DA-5512 on the Viability and Proliferation of hDPCs

**Figure 1:**
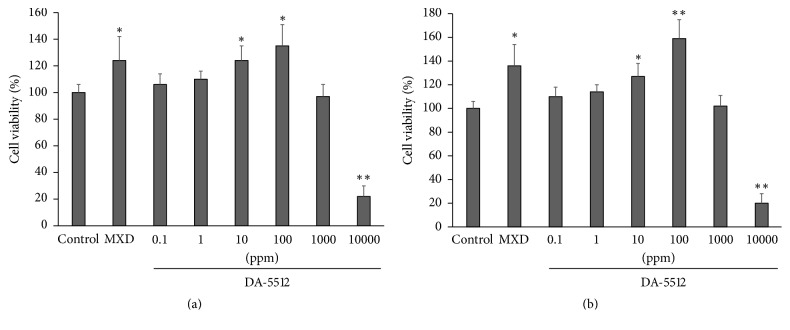
Viability of human dermal papilla cells after (a) 24 h and (b) 48 h treatment with various concentrations of DA-5512. Cell viability (%) = (mean test absorbance)/(mean control absorbance) × 100. Concentration of minoxidil (MXD) was 1 *μ*M. All values are expressed as the mean ± SD (*n* = 8). ^*∗*^*p* < 0.05 and ^*∗∗*^*p* < 0.01 versus control, respectively.

**Figure 2:**
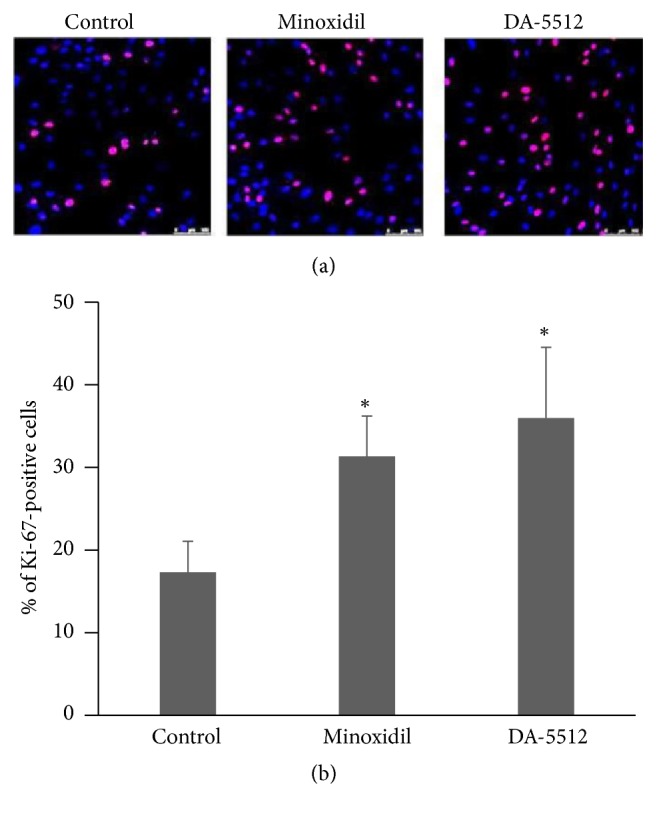
Effects of DA-5512 on Ki-67 levels in human dermal papilla cells. (a) The number of proliferating (Ki-67-positive, red) cells versus total hDPCs was determined. Nuclei were stained with DAPI (blue). Scale bar = 100 *μ*m. (b) Ki-67-positive cells were counted in four nonoverlapping fields in each group and normalized to the total number of hDPCs (DAPI-positive). The concentrations of MXD and DA-5512 were 1 *μ*M and 100 ppm, respectively. All values are expressed as the mean ± SD. ^*∗*^*p* < 0.05 versus control.

DA-5512 and MXD (1 *μ*M) significantly enhanced the viability of hDPCs compared with the control group (each *p* < 0.05) (Figure 1). Further, 10,000 ppm of DA-5512 was cytotoxic (*p* < 0.01). We found that 100 ppm of DA-5512 and 1 *μ*M MXD significantly increased the number of Ki-67-positive cells compared with the control group (*p* < 0.05) (Figure 2).

### 3.2. DA-5512 Promotes the Growth of the Hair of C57BL/6 Mice

**Figure 3:**
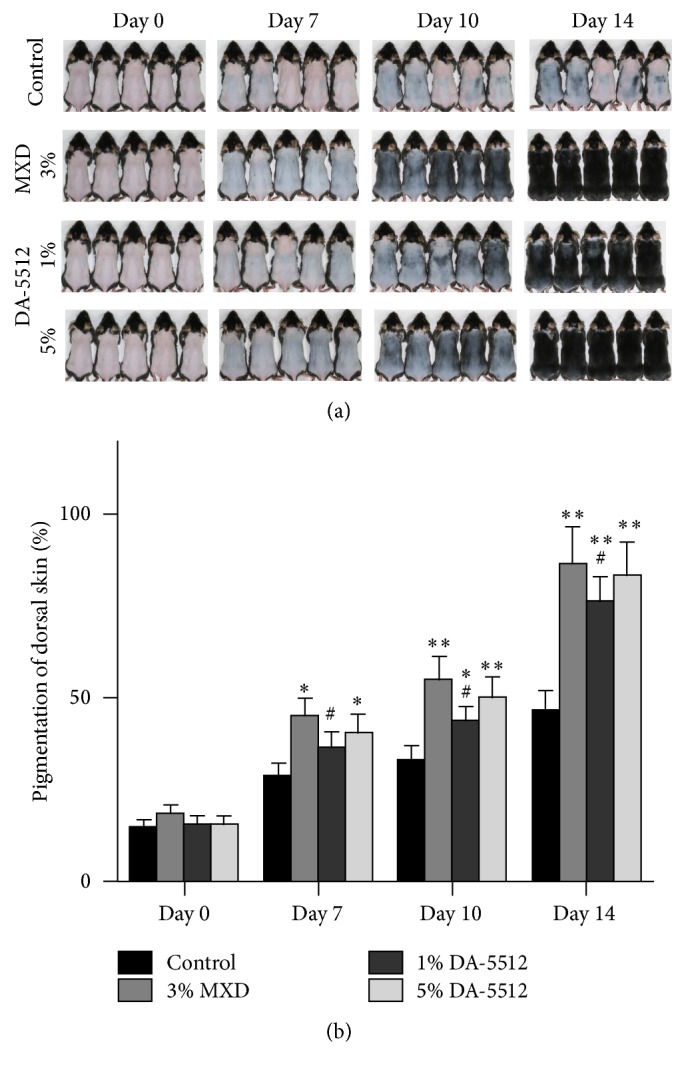
Hair-growth-promoting effects of DA-5512 on C57BL/6 mice. (a) Photographs of mice over 14 days of treatment with control (control, topical), 3% topical MXD, and 1% or 5% topical DA-5512. (b) Quantified pigmentation of the dorsal skin (%) in C57BL/6 mice over 14 days of treatment. ^*∗*^*p* < 0.05 and ^*∗∗*^*p* < 0.001 versus control, and ^#^*p* < 0.05 versus 3% MXD.

**Figure 4:**
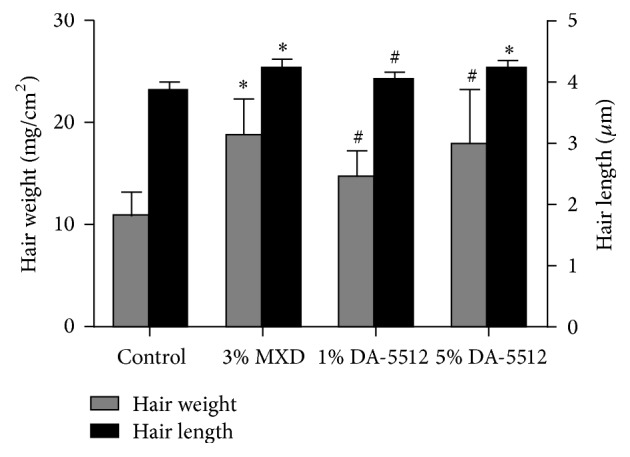
Hair length and weight of the dorsal skin of C57BL/6 mice after 14-day treatment. All values are expressed as the mean ± SD. ^#^*p* < 0.05 and ^*∗*^*p* < 0.01 versus the control.

Figure 3 demonstrates the hair-growth-promoting effects of DA-5512 on C57BL/6 mice after 14 days of treatment. NC-group mice exhibited only a faint appearance of hair after treatment for 14 days. The groups treated with PC (3% MXD) and DA-5512 showed significant hair growth, which occupied the major portion of the back (Figure 3(a)). Digital image analysis of hair growth showed significantly higher values for the DA-5512-treated groups compared with that of the NC group and similar values compared with those of the PC group (*p* < 0.05) (Figure 3(b)). Notably, the 5% DA-5512 dose group showed increased hair growth compared with that of the 1% DA-5512 dose group. The mean hair lengths and weights on day 14 are shown in Figure 4. The DA-5512 and the 3% MXD groups exhibited significant increases in mean hair length and weight on day 14 (*p* < 0.05). There were no significant differences in the measurements of hair weight and length between the 3% MXD and 5% DA-5512 groups.

**Figure 5:**
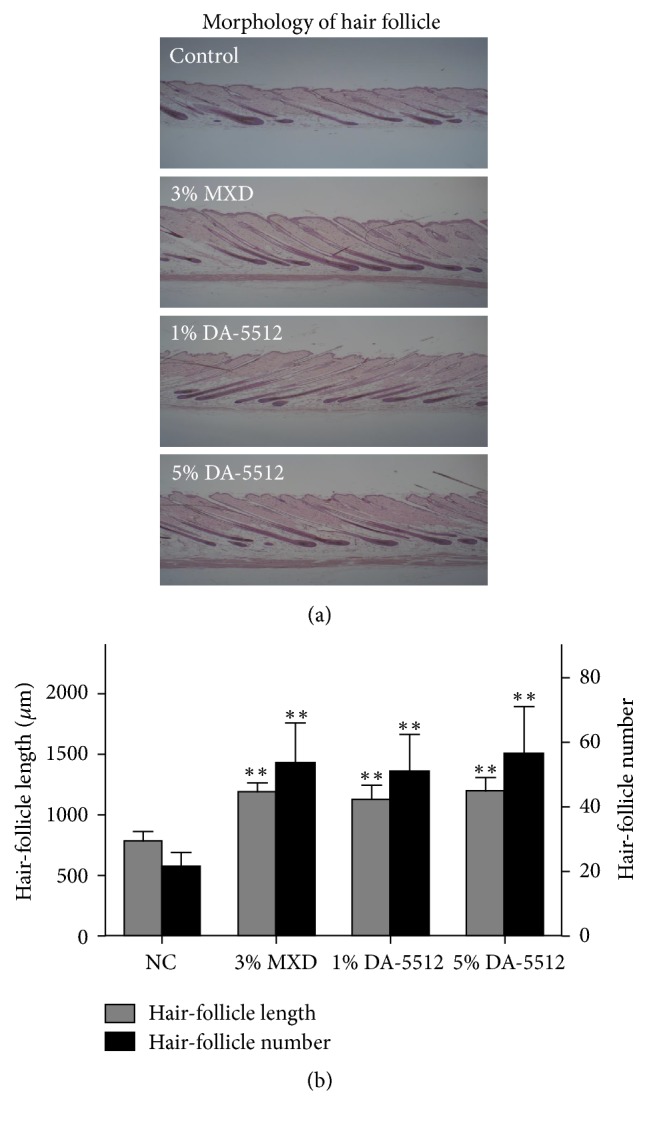
Histological analysis (H&E staining) of the basal layer of the epidermis of C57BL/6 mice after 14 days of treatment. (a) Morphology and (b) length and number of hair follicles in the epidermis. Digital images were obtained using a light microscope (100× magnification). All values are expressed as the mean ± SD. ^*∗∗*^*p* < 0.001 versus control.

To confirm the effect on the hair-growth cycle following the application of DA-5512, we analyzed the follicular morphology of the dorsal skin.  Figure 5 demonstrates the morphology, mean HF count, and mean HF length of each group. HF lengths and numbers of the 3% MXD and DA-5512 groups differed significantly from those of the NC group (*p* < 0.001) (Figure 5(b)). Mice given DA-5512 had significantly greater numbers of HFs compared with those of mice given the vehicle, but not compared with those of mice receiving 3% MXD.

### 3.3. Stimulation of Hair Growth and Promotion of Hair Health by DA-5512 in Patients with Alopecia

#### 3.3.1. Age and Sex of the Study Subjects

Each of the three groups comprised at least 10 subjects. Two participants each from the DA-5512 and placebo groups and three participants from the 3% MXD group resigned before completion of the study, and 23 participants completed the experiment. The percentages of men and women were 17.39% (4/23) and 82.61% (19/23), respectively. In the placebo and DA-5512 groups, the percentages of men and woman participants were 25% (2/8) and 75% (6/8), respectively. The percentages of men and women in the 3% MXD group were 0% (0/7) and 100% (7/7), respectively. There were no significant differences in the ages (years) of the three groups (DA-5512, 49.25 ± 6.41; 3% MXD, 49.00 ± 8.70; placebo, 50.00 ± 5.61).

#### 3.3.2. Hair Density and Hair-Shaft Diameter

**Figure 6:**
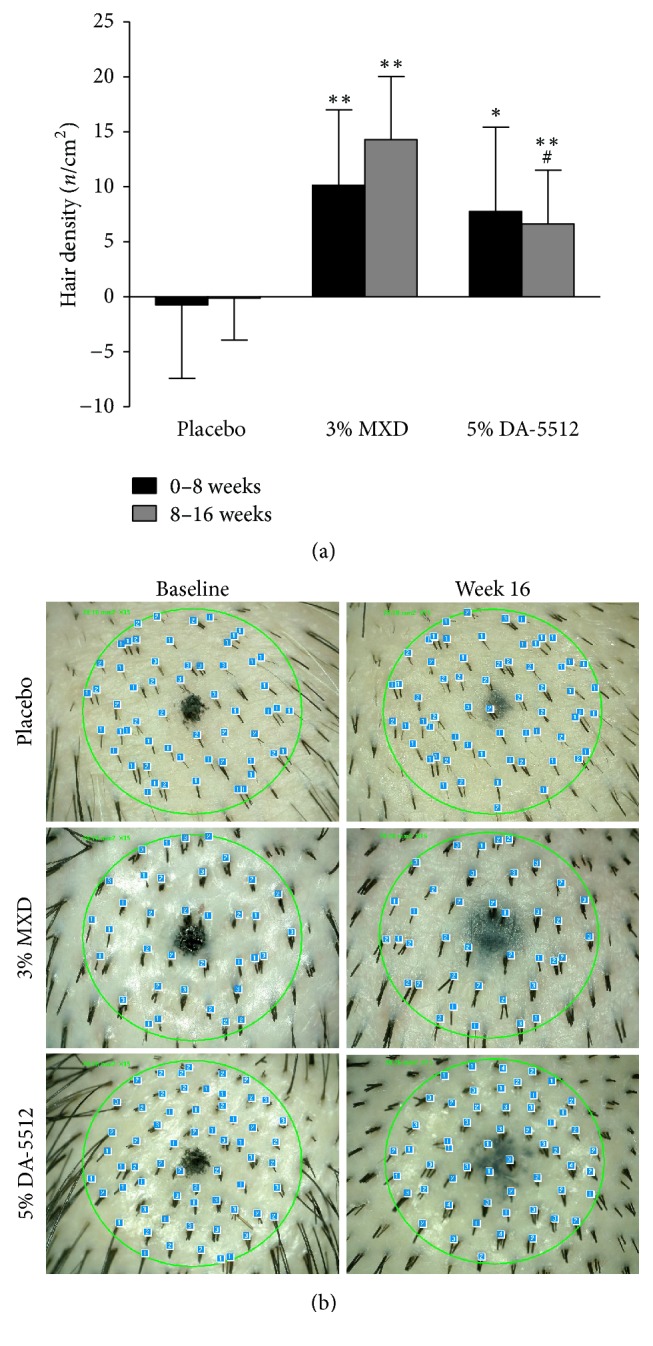
(a) Changes in hair density through days 0–8 and 8–16 days after treatment with placebo, 3% MXD, or 5% DA-5512. All values are expressed as the mean ± SD. ^*∗*^*p* < 0.05 and ^*∗∗*^*p* < 0.01 versus placebo, and ^#^*p* < 0.05 versus 3% MXD. (b) Folliscopy of hair density at baseline and week 16 after treatment with placebo, 3% MXD, or 5% DA-5512.

**Figure 7:**
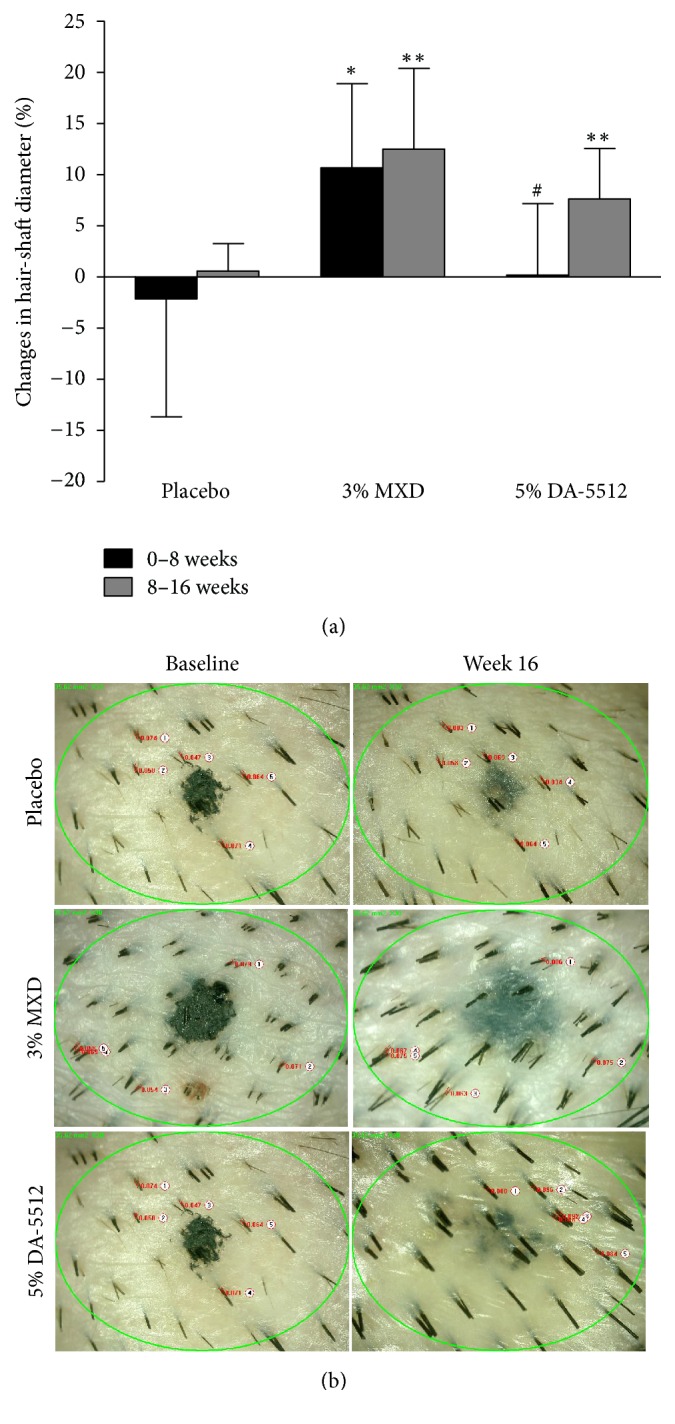
(a) Changes in hair-shaft diameter through days 0–8 and 8–16 after treatment with placebo, 3% MXD, or 5% DA-5512. All values are expressed as the mean ± SD. ^*∗*^*p* < 0.05 and ^*∗∗*^*p* < 0.01 versus placebo, and ^#^*p* < 0.05 versus 3% MXD. (b) Folliscopy of hair-shaft diameters at baseline and after 16 weeks of treatment with placebo, 3% MXD, or 5% DA-5512.

The changes in hair density and hair-shaft diameter determined using folliscope measurements over 16 weeks are shown in Figures 6 and 7, respectively. The mean changes in hair density (*n*/cm^2^) after 8 and 16 weeks of treatment with 3% MXD (10.14 ± 6.84 and 14.29 ± 5.74) and 5% DA-5512 (7.75 ± 7.66 and 6.62 ± 4.89) were significantly higher (*p* < 0.01) compared with those treated with placebo (−0.75 ± 6.67 and −0.125 ± 3.81). Similarly, the mean changes in hair-shaft diameter (mm) after 8 and 16 weeks of treatment with 3% MXD (0.007 ± 0.006 and 0.009 ± 0.006, resp.) were significantly higher (*p* < 0.05) compared with those of the placebo group (−0.002 ± 0.009 and 0.000 ± 0.002). However, the mean changes in hair-shaft diameter after 8 weeks of treatment with 5% DA-5512 (0.000 ± 0.006) were not significant, while those after 16 weeks of treatment with 5% DA-5512 (0.006 ± 0.003) were significantly higher (*p* < 0.05) compared with those of the placebo group. The mean changes in diameter following treatment with 3% MXD were significantly higher compared with treatment with 5% DA-5512 after 8 weeks (*p* < 0.05), although the difference was not statistically significant after 16 weeks.

#### 3.3.3. Hair-Growth Rate

**Figure 8:**
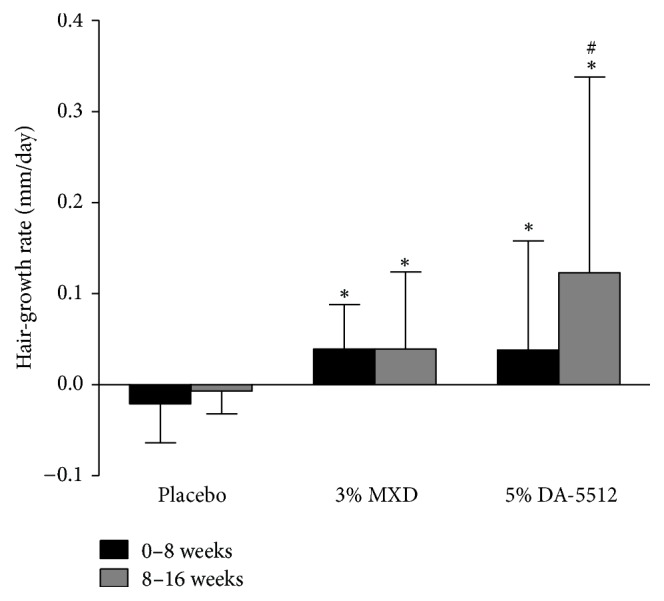
Changes in hair-growth rate through days 0–8 and 8–16 after treatment with placebo, 3% MXD, or 5% DA-5512. All values are expressed as the mean ± SD. ^*∗*^*p* < 0.05 versus placebo. ^#^*p* < 0.05 versus 3% MXD.

The mean hair-growth rates (mm/day) of five strands of hair in the target area after 8 and 16 weeks of treatment with 3% MXD and 5% DA-5512 are shown in Figure 8. The rates after 8 and 16 weeks after treatment with 3% MXD (0.039 ± 0.049 and 0.039 ± 0.085, resp.) and with 5% DA-5512 (0.035 ± 0.078 and 0.134 ± 0.174, resp.) were significantly higher (*p* < 0.05) compared with those of the placebo group (−0.021 ± 0.043 and −0.007 ± 0.025). The performance of 5% DA-5512 was superior to that of 3% topical MXD after 16 weeks (*p* < 0.05).

#### 3.3.4. Investigator's Assessment Score of Hair Density

**Figure 9:**
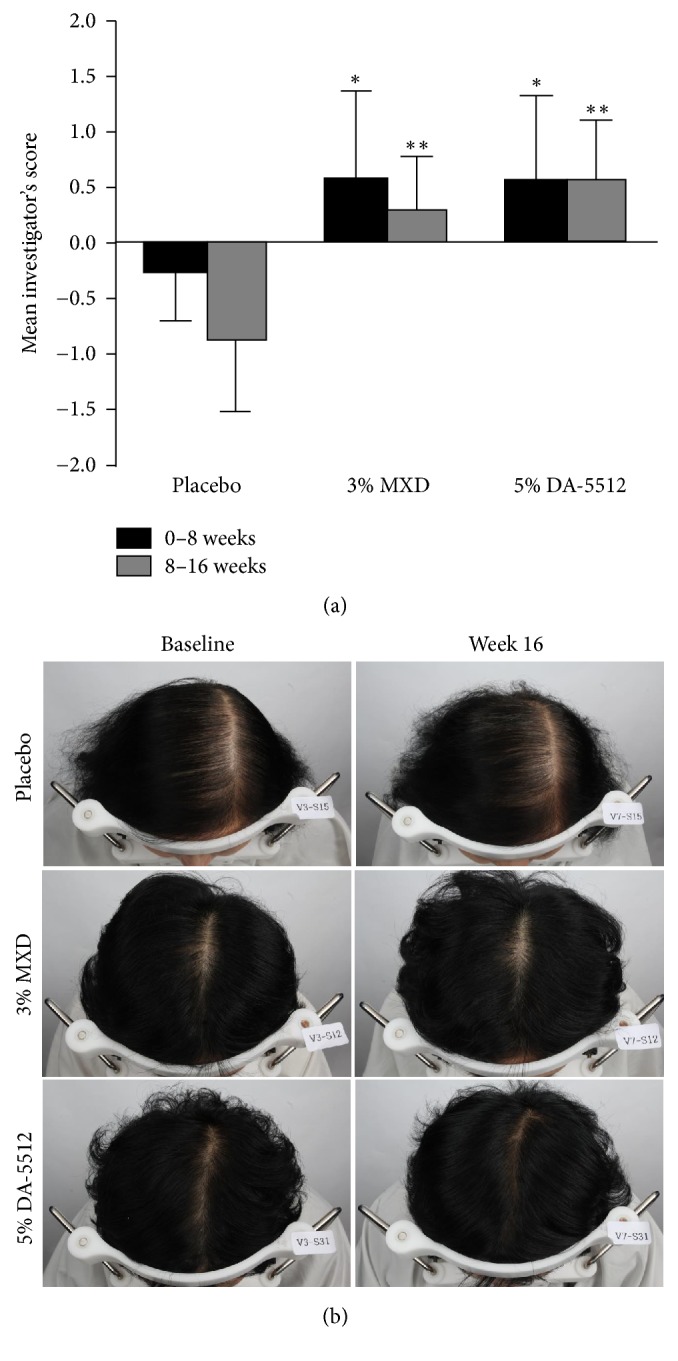
(a) Investigator's assessment score through days 0–8 and 8–16 after treatment with placebo, 3% MXD, or 5% DA-5512. All values are expressed as the mean ± SD. ^*∗*^*p* < 0.05 and ^*∗∗*^*p* < 0.01 versus placebo. (b) Representative photographs of patients at baseline and week 16.

An investigator, uninformed of the nature of the study, assessed hair density using a standardized 7-point mean score. As shown in Figure 9, the mean scores for hair densities of the 3% MXD group (each 0.571 ± 0.787, 0.286 ± 0.488) and the 5% DA-5512 group (each 0.500 ± 0.756, 0.500 ± 0.535) were significantly higher compared with those of the placebo group (each −0.250 ± 0.463, −0.875 ± 0.641) after 8 (*p* < 0.05) and 16 weeks (*p* < 0.01), respectively. Most subjects in the 3% MXD and 5% DA-5512 groups exhibited slight or moderate improvement in hair-density assessment scores.

#### 3.3.5. Clinical Response

Clinical responses were assessed via an investigator's assessment and each patient's self-assessment of the efficacy of the treatment according to hair growth, hair density, or satisfaction according to a 10-point scale, and each patient's self-assessment of scalp condition used a standard 5-point rating scale (Table 2). No significant differences were observed among the three groups at the end of week 16. The self-assessment scores of the subjects' scalp conditions were based on improvement of horny skin, itching, excess sebum, and dandruff, and the satisfaction scores showed no significant differences between the three groups.

#### 3.3.6. Safety Evaluation

No product-related adverse effects were exhibited by subjects who received topical 5% DA-5512, 3% MXD, or placebo (data not shown).

## 4. Discussion

The HF is a sac or a structure in the skin, which produces hair through a three-phase growth cycle as follows: anagen, catagen, and telogen [30]. HF morphogenesis involves the downward migration of bulge stem cells and their entry into the matrix where they proliferate and differentiate to form HFs [31]. During anagen, follicles are long and straight and increase in size and number to produce an entire hair shaft. During catagen and telogen, the follicles reset and generate stem cells. When stem cells in the HFs are activated in response to the signal to start the next growth phase, they start a new anagen phase and produce a new hair shaft [32]. This process is clinically important, because many patients with hair disorders suffer from undesired alterations in HF cycling [33].

Cultured normal hDPCs, a hair-follicle organ culture model, and hair and skin specimens of mice were used to determine the effects of DA-5512. DA-5512 concentrations from 10 to 100 ppm enhanced the viability of hDPCs; however, >10,000 ppm was toxic. High concentrations overstimulate hair-follicle metabolism to cause extensive consumption of energy reserves, exhaustion of proliferative capacity, and inhibition of hair-shaft elongation. Further, the mechanism responsible for the stimulatory effects of DA-5512 on hair-follicle growth is unknown. To investigate the proliferative activity of hDPCs in response to DA-5512, we chose a dose of 100 ppm. We found that Ki-67 levels increased in hDPCs upon treatment with DA-5512 or MXD. Ki-67, which detects nuclei in matrix keratinocytes, is an indicator of the proliferation of anagen hair follicles [34, 35]. Therefore, DA-5512 may promote hair regrowth, because it increased the proliferation of hDPCs.

C57BL/6 mice are used to screen for the effects of hair-growth-promoting agents [36]. C57BL/6 mice lack melanocytes in their skin, and therefore melanogenesis occurs only in hair follicles, and melanins are produced only during anagen phase [37]. Therefore, melanogenesis is strongly related to the hair-growth cycle in these mice. The skin of C57B1/6 mice is pale pink in the resting telogen phase, becomes dark gray or black during active hair growth in the anagen phase, and returns to pale pink in the catagen phase when hair growth ceases and the skin transitions back to the telogen phase. Hair growth of the DA-5512-treated group was higher compared with that of the NC group and was similar to that of the 3% MXD-treated group. DA-5512 significantly shortened the time for skin darkening and hair growth and increased hair length and weight, indicating that DA-5512 stimulated the anagen phase of the hair-growth cycle. Histological analysis revealed that the number of hair follicles in the DA-5512-treated group was significantly higher compared with that of the NC group, suggesting that DA-5512 may elicit its hair-growth-promoting activity through an increase in hair-follicle number and through stimulation of anagen.

We conducted a double-blind, placebo-controlled, randomized clinical trial to evaluate the ability of DA-5512 to promote hair growth in patients with pattern hair loss. Statistically significant increases in hair density, hair diameter, hair-growth rate, and investigator's assessment scores of hair density characterized the 3% MXD and 5% DA-5512 groups compared with those of the placebo group after 16 weeks. Our data reveal that topical treatment with 5% DA-5512 led to increases in hair density and diameter, which was significantly greater at 16 weeks compared with 8 weeks, suggesting that using DA-5512 continuously for longer times may have beneficial effects on hair loss.

Hair diameter and density tend to decrease with hair loss, suggesting that because DA-5512 treatment increased hair diameter and density, it may enhance hair growth or slow the progression of hair loss. Interestingly, the patient's self-assessment scores did not demonstrate a significant outcome for patterned hair loss. However, an overall improvement in hair growth or density, in scalp condition (horny skin, itching, excess sebum, and dandruff), and in satisfaction with 5% topical DA-5512 compared with placebo indicated a definite advantage of DA-5512 for treating patterned hair loss.

Several varieties of plants are used to prepare DA-5512 to prevent or treat hair loss, to nourish hair, and to improve the esthetic properties of hair. These plants produce inhibitors of 5*α*-reductase activity. Green tea is rich in the flavanol group of polyphenols called catechins, and green tea polyphenols positively affect hair growth and follicle health. Possible mechanisms of action include inhibition of apoptosis, radioprotection of follicle cells, antioxidant activity, and potential follicular inhibition of TGF-*β*1 [38].

Green tea is a popular herbal remedy that contains catechins that may inhibit 5*α*-reductase. EGCG, the most abundant catechin found in green tea, prevents or treats androgenetic alopecia by selectively inhibiting 5*α*-reductase activity [39].* P. emblica* L. and* Zingiber officinale* promote hair growth by inhibiting 5*α*-reductase activity [19] in vitro and in vivo. Moreover,* Puerariae flos* inhibits testosterone 5*α*-reductase in vitro and exhibits antiandrogenic activity that was detected using a hair-growth assay in testosterone-sensitive male C57Black/6NCrSlc mice [40]. 5*α*-Reductase catalyzes the conversion of testosterone into dihydrotestosterone (DHT) that combines with the same androgenic receptor to form a conjugate, which leads to hair-follicle miniaturization and then to telogen [41]. Thus, DA-5512 might influence hair growth by inhibiting 5*α*-reductase activity.

A preclinical study using a* Pueraria thunbergiana* (pine) needle extract revealed its role in the improvement of hair growth in C57BL/6 mice [42]. This study shows that pine needle extract treatment increases the levels of IGF-1 and VEGF, which play a role in increasing the size of hair follicles, in angiogenesis in the outer root sheet, and inhibit TGF-*β*1, which disrupts hair growth. High levels of TGF-*β*1 stimulate androgen or DHT production, which inhibits the growth of keratinocytes in dermal papilla. Pine needle extract is an antioxidant that is a potent scavenger of free radicals. Further, it inhibits the production of reactive oxygen species by stimulating superoxide dismutase and glutathione peroxidase to promote hair growth and to treat hair loss.

The antihypertensive effects of the* T. terrestris* fruit are similar to those of MXD, and its beneficial effects are attributed to vasodilator activity [25]. The mechanism of the effect of* T. terrestris* fruit on human hair growth is unknown, but it may act as a growth factor for hair roots. Therefore, these properties suggest that the various components of DA-5512 might account for its hair-growth-promoting activity that enhances hair health.

In summary, we show here that in a mouse model DA-5512 promoted the proliferation of human DPCs and stimulated hair growth. Further, a clinical study detected enhanced hair growth or slowed progression of hair loss upon treatment of patients with DA-5512. However, the mechanisms of DA-5512 associated with its effect of hair loss remain to be determined and therefore require further study.

In conclusion, the natural components of DA-5512 might influence hair-growth-promoting activity and enhance hair health and can therefore be considered an effective option for treating hair loss.

## Figures and Tables

**Table 1:** Plants used to prepare herbal extracts of the proprietary formulation DA-5512.

Subject	Custom	Identifier	Local area	Nation
*Thea sinensis* L.	Soa Dawon herb	Myung-hae Choi	Bosung Jeonnam	Korea
*Tribulus terrestris*	Hong-il dang	CK Trading Company	North India Punjab	India
*Pinus densiflora*	The one herb	Hong-geun Kim	Chuncheon, Gangwon-do	Korea
*Emblica officinalis*	Duc-su mu yuck	Bayir Chemical Company	Bangalore	India
*Pueraria thunbergiana*	The one herb	Hansin Logis Company	Guangxi Zhuangzu	China
*Zingiber officinale*	The one herb	Chunha Company	Shanding	China

**Table 2:** Investigator's assessment and patients' self-assessment of clinical responses.

End point	Group		
	Placebo	3% MXD	5% DA-5512
*Efficacy assessment scores* ^*(1)*^			
Hair growth	6.875 ± 1.553	6.857 ± 1.574	6.250 ± 1.282
Hair diameter	6.375 ± 1.598	5.143 ± 1.574	5.750 ± 1.389

*Satisfaction* ^*(2)*^			
Hair line styling	6.500 ± 1.604	5.429 ± 0.787	5.750 ± 1.389
Hair condition	5.875 ± 2.900	5.857 ± 1.574	5.750 ± 2.816
Hair product	6.375 ± 2.264	5.571 ± 1.272	5.875 ± 1.356

*Scalp condition* ^*(3)*^			
Keratin	1.375 ± 0.744	1.857 ± 0.690	1.625 ± 0.916
Pruritus	1.785 ± 0.164	2.000 ± 0.816	1.500 ± 1.195
Sebum	1.378 ± 0.954	1.857 ± 0.900	1.875 ± 0.641
Dandruff	1.125 ± 0.991	1.429 ± 0.787	1.250 ± 1.035

^(1) (2)^A standard 10-point rating scale of investigator's and patients' assessments from 0 (worse) to 10 (great) on visual scales. ^(3)^A standard 5-point rating scale of patients' assessments from 0 (greatly decreased) to 5 (greatly increased) on visual scales. All the values are expressed as the mean ± SD.
